# The Burden of Preoperative Stress: Biological Mechanisms and Postoperative Outcomes

**DOI:** 10.3390/brainsci16020219

**Published:** 2026-02-11

**Authors:** Aaqib Syed, Tallita Menezes, Andrew Bisenius, Aleksandar Sic, Nebojsa Nick Knezevic

**Affiliations:** 1 Department of Anesthesiology, Advocate Illinois Masonic Medical Center, Chicago, IL 60657, USA; aaqib.syed165@gmail.com (A.S.); tallita.menezes@aah.org (T.M.); andrew.bisenius@aah.org (A.B.); aca.smed01@gmail.com (A.S.); 2 Chicago Medical School, Rosalind Franklin University of Medicine and Science, North Chicago, IL 60064, USA; 3 Department of Anesthesiology, University of Illinois, Chicago, IL 60612, USA; 4 Department of Surgery, University of Illinois, Chicago, IL 60612, USA

**Keywords:** preoperative stress, HPA axis, cortisol, surgical stress response, postoperative outcomes, perioperative anxiety, inflammation, hyperglycemia, postoperative infections, pain modulation

## Abstract

Preoperative psychological stress is a highly prevalent but mostly underrecognized factor influencing perioperative physiology and postoperative outcomes. This narrative review synthesizes current evidence on the known mechanisms connecting preoperative stress to adverse surgical outcomes, with particular emphasis on HPA axis functioning, cortisol dynamics, inflammatory signaling and pain modulation. Elevated preoperative anxiety affects a substantial proportion of surgical patients and is consistently associated with increased analgesic and anesthetic requirements, higher postoperative pain intensity, greater risk of chronic postsurgical pain, neuropsychiatric complications, metabolic dysregulation and postoperative infections. Stress-related elevations in cortisol and pro-inflammatory cytokines, particularly interleukin-6, appear to mediate these effects through interactions with immune, metabolic, and central nervous system pathways. Stress-related pain modulation is reflected not only in experimental models but also in clinically measurable outcomes, underscoring its relevance for perioperative care. Despite growing recognition of these associations, standardized strategies for integrating stress assessment and biomarkers into perioperative risk stratification remain limited. Given that preoperative stress is potentially modifiable, targeted psychological, analgesic, and metabolic interventions may represent valuable opportunities to improve recovery, reduce complications, and prevent pain chronification.

## 1. Introduction

Stress is the human’s response to disrupted homeostasis. Stress factors can include external stressors (environmental, psychological, or social) and internal stressors (illness, medical procedure, or anxiety) [1,2]. When exposed to stress, a complex stress response is initiated including the HPA axis, sympathetic–adreno–medullary (SAM) axis and immune system which, together, help the body prepare for stressors [3]. Among these, preoperative anxiety has drawn considerable attention due to its impact on postoperative outcomes.

Affecting an estimated 60–80% of surgical patients, preoperative stress is a highly prevalent yet underrecognized factor that influences surgical recovery [4,5]. Patients experiencing clinically significant preoperative anxiety have been reported to require higher anesthetic and analgesic doses, and appear to have higher rates of postoperative delirium [5]. These patients also have delayed achievement of recovery milestones such as a modified Aldrete score of 9, resulting in extended stays in the post-anesthesia care unit (PACU). Consistent with this, further research suggests a relationship between preoperative anxiety and postoperative adverse events, including sleep disturbances, increased pain with nausea and vomiting, and neurocognitive dysfunction, although causal inference remains limited [6].

While these relationships are well documented, the underlying biological mechanisms connecting stress to surgical outcomes remain insufficiently explored. This review therefore explores the molecular and clinical intersection between preoperative stress and postoperative outcomes.

## 2. Methodology

A literature search was performed in PubMed/MEDLINE, Scopus, and Web of Science for peer-reviewed articles published up to November 2025. Search terms included combinations of keywords: preoperative stress, perioperative anxiety, surgical stress response, HPA axis, cortisol, inflammation, postoperative pain, and postoperative outcomes. Articles were selected based on their relevance to the topic, methodological quality, and contribution to mechanistic, clinical, or translational understanding. Both experimental and clinical studies were considered. Editorials, conference abstracts, and studies lacking sufficient methodological detail were excluded, unless they provided highly relevant or seminal conceptual, mechanistic, or clinical insights directly pertinent to the scope of this review. Additional relevant articles were identified through the manual screening of reference lists. Evidence was synthesized qualitatively to highlight consistent mechanisms, areas of agreement, and gaps in knowledge. Given the narrative nature of the review, no formal risk-of-bias assessment or quantitative synthesis was performed.

## 3. Different Stress Responses

The stress response is basic prerequisite for the body to adapt to changes in the environment and maintain the organism’s homeostasis. A stressor is any factor that unbalances the organism and can be classified as chronic, such as emotional strain, or acute, such as tissue injury [7]. The body responds differently to different stressors. The brainstem and hypothalamus are the main regions for processing physical stressors. Initially mediated by the sympathetic-adrenal (SAM) system, it generates rapid physical responses that make an immediate reaction to the threat possible. On the other hand, psychological stressors require physical and cognitive responses and, therefore, are processed in a more complex way, recruiting complex limbic circuits in addition to basic autonomic pathways [8].

Chronic or acute exposure also leads to different responses. One of the mechanisms behind those in acute and chronic stress is resistance of glucocorticoid receptors caused by its chronic activation, thus causing dysregulation of the HPA axis [9,10]. Individuals under chronic stress may exhibit synaptic remodeling due to alterations in glutamatergic and GABAergic pathways in the hippocampus, leading to mood and memory changes [11]. Consistently elevated levels of glucocorticoids also promote the expression of different genes, activating an inflammatory response [12].

This physiological difference between chronic and acute stress can be partially explained through the allostatic load. Allostatic load is the cumulative biological overload after consecutive attempts to adapt to demands [13]. This increase generates a dysregulation of the body’s adaptive stress systems [14]. As a consequence, an increased allostatic load has been correlated with poor health, physical function [15] and mental health [16].

In the perioperative setting, both acute surgical trauma and chronic psychological stress contribute to dysregulation of these adaptive systems [17]. Acute stress begins after tissue damage and leads to a response from the organism characterized by sterile inflammation and metabolic and neuroendocrine dysregulation [18]. This response can be influenced, for example, by the type of anesthetic or surgical technique, but in isolation they do not alter postoperative morbidity and mortality [19]. On the other hand, preoperative psychological interventions can influence biochemical and neuroendocrine responses to surgical stress and may be associated with improvements in postoperative outcomes [20].

## 4. HPA Axis in the Surgical Stress Response

The human capacity to adapt to stressors is an essential mechanism for the survival of the species and allows the organism to maintain homeostasis in the face of challenges, whether physical or psychological [21]. The hypothalamic–pituitary–adrenal axis stimulation is believed to be the central mechanism involved in the response, stimulating the release of these glucocorticoids, which provide energy to handle the threat, whether real or perceived [22].

**Figure 1:**
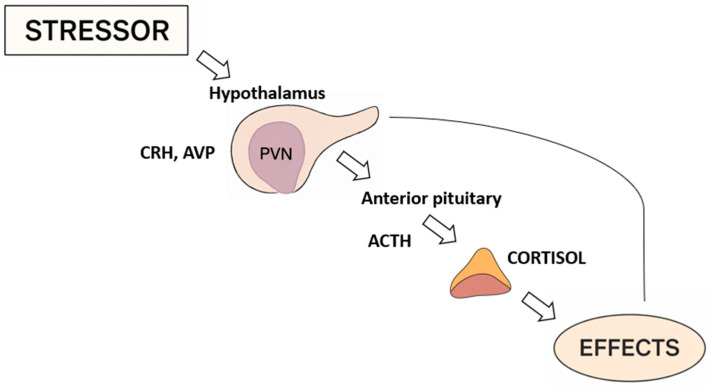
Activation of the HPA axis in response to stress. Made by authors in BioRender online application, available at: https://www.biorender.com/ (accessed on 4 December 2025).

Following exposure to stressors, neurons in the paraventricular nucleus (PVN) [23], release the corticotropin-releasing hormone (CRH) and vasopressin to portal circulation. These hormones stimulate the anterior pituitary gland to secrete the adrenocorticotropic hormone (ACTH) [24]. The adrenocorticotrophic hormone then stimulates the adrenal cortex which leads to the production of glucocorticoids [23]. Cortisol is then secreted in a pulsatile manner; just this rhythmic release is fundamental to maintaining cellular responsiveness and ensuring the multiple effects of glucocorticoids [25,26]. The HPA system is characterized by activity depending on the circadian rhythms or the ultradian rhythms, according to the pulsatile release of the cortisol into bursts of activity throughout the daytime hours [27,28]. An overview of the HPA axis response to stressors, as well as its downstream hormonal effects, is summarized in Figure 1.

This activation of the HPA neuroendocrine cascade can be started by a lot of factors, which can be physical, (direct tissue injury for example), or psychological (involving the perception of a threat) [29]. Among physical stressors, surgical trauma induces a particularly intense and coordinated cascade of hormonal and metabolic changes [30]. Postsurgical response of the body is a complex neuroendocrine and metabolic process, with an inflammatory/immune response involved. Briefly, there is growth factor/energy substrate release, as well as of inflammatory mediators, inhibition of anabolic hormones and sodium and fluid retention [18,31].

The activation of the HPA axis represents a central component of the surgical stress response and provides a framework for the downstream metabolic alterations. However, most perioperative studies linking HPA axis activity to postoperative outcomes remain observational, and direct evidence that modifying the HPA axis improves clinical outcomes is limited [18]. Prospective trials that integrate standardized stress measures with repeated neuroendocrine sampling and postoperative outcomes are needed to understand the clinical relevance of perioperative HPA axis modification.

## 5. Cortisol: From Intracellular Signaling to Systemic Responses

The final hormone released from the HPA axis is cortisol, a steroid-structured hormone made from cholesterol. More than 95% of cortisol is attached to corticosteroid-binding globulin into the circulation. The fraction of bioavailable free cortisol can be influenced by many factors such as body temperature or inflammation [32]. The cortisol target of action is located at the intracellular level. Upon binding to cortisol, the intracellular receptor detaches from proteins such as hsp90 and translocates to the cell nucleus, where it connects to specific regions of DNA called glucocorticoid-response elements. This can alter the expression of several genes. Furthermore, GR is also involved in the regulation of activity of other transcription factors, including AP-1, NF-κB, influencing the inflammatory activity and stress responses [33].

It also crosses the blood–brain barrier and binds to mineralocorticoid (MR) and glucocorticoid (GR) receptors in the central nervous system [34]. The MR are highly expressed in cortical and limbic areas including the hippocampus, the amygdala, and medial prefrontal cortex [35], while GR are distributed widespread but with lower affinity. The role of these receptors is categorized into the fast non-genomic response, followed by the slow genomic response, involved in regulating neuronal excitability, energy, defensive responses, emotional, motivational, and social processes [36]. A number of studies have also explored the impact of cortisol levels on cognitive functions, with particular attention to working memory and attention capabilities [37,38].

Glucocorticoids also affect blood pressure; this is due to the expression of the enzyme phenylethanolamine-N-methyltransferase, which converts norepinephrine into epinephrine in the adrenal medulla [39]. Cortisol also plays a role in the immune system, where it acts by reducing cytokine production and the inflammatory response during the infection or inflammation process, but also in response to non-immunological stressors, such as physical or psychological stress [40]. Ultimately, another important glucocorticoids effect is the breakdown of glycogen and gluconeogenesis, which helps to meet the energy demands resulting from stress [41].

## 6. Cortisol as an Indicator of Clinical Risk

Cortisol is used effectively as an indicator to measure the degree of the stress response reaction. To monitor its levels, the options available are hair, saliva, and urine samples [42]. Cortisol measured through hair samples is a good long-term biomarker because it reflects prolonged exposure to the hormone [43]. Hair cortisol levels are also linked with the fear and anxiety levels in children [44]. In adults and the elderly, hair cortisol levels increase with factors such as age, alcohol consumption, and smoking, correlating with a higher risk of stress-related illnesses [45]. Overall, hair cortisol is considered the most useful and validated marker for assessing chronic stress in different populations.

Salivary cortisol has been consistently studied as a biomarker. Individuals with stress or burnout had elevated levels of cortisol in their saliva compared to those without stress or burnout, particularly one hour upon waking [46]. The application of salivary cortisol levels as a stress and depression indicator is still worthy of more study [47]. There are also high levels of heterogeneity, according to the lack of standardization in the kits used, the nature of the populations, and the possible risk factor differences [48]. The clinical utility of salivary cortisol depends on improved methodological standardization and greater awareness of confounding factors, including psychological stress, sampling conditions, and timing relative to the stimulus [49]. Despite these limitations, evidence supports the physiological validity of salivary cortisol as a noninvasive stress biomarker. There is a proven strong positive correlation between plasma and salivary cortisol levels [50].

Cortisol can also be used to assess the effect of pharmacotherapy on mental illness by comparing levels collected before and after therapy [51,52].

Cortisol is also involved in the evaluation of cardiovascular risk. Plasma cortisol levels in the morning are positively related to glucose levels, blood pressure, and other markers of cardiovascular risk, and also with subclinical markers of atherosclerosis, including carotid plaques and coronary calcification, among others [53]. Cortisol levels can be found to be significantly elevated in patients months before a cardiac event such as acute myocardial infarction [54]. However, the evidence linking cortisol directly to clinical cardiovascular events is still limited and inconclusive. A prospective cohort study was carried out on 3052 patients who underwent coronary angiography by Pilz et al.in 2021 [55]. Despite the link between cortisol and the presence of an unfavorable profile of cardiovascular risk, cortisol was neither recognized as a significant independent predictive factor for total or cardiovascular mortality, adjusting for risk factors in the study by Pilz et al. [55]. Elevated levels of urinary stress hormones, including cortisol, are connected with a higher risk of hypertension in previously healthy population [56].

A secondary analysis [57] evaluated the multicenter randomized controlled EFFORT study, consisting of 764 hospitalized patients who are also at risk of malnutrition. The mean cortisol levels on admission were higher in those with higher levels of malnutrition risk (NRS ≥ 5) and those who reported lower appetite. Those with cortisol levels in the highest quartile had higher mortality risk at 30 days and 5 years. The study provided insights that cortisol levels can be used as markers of risk or response to treatment in multimorbid hospitalized patients with malnutrition.

In perioperative medicine, the importance of using stress markers lies in the possibility of identifying patients who will benefit from delaying surgery for optimization or who may require close patient monitoring [58,59]. Multimodal and multidisciplinary interventions, including psychological interventions, can contribute to a better response to surgical stress and reduce postoperative complications [60].

Cortisol levels are currently measured by mass spectrometry or immunoassay, requiring a complex laboratory setup and trained professionals. This translates into a time-consuming, expensive, and inefficient process for monitoring the dynamic rhythm of hormone secretion [60]. Science is moving towards the possibility of a continuous monitoring system [61,62], including wearable systems for continuous cortisol monitoring, integrating sensors and algorithms [63].

Cortisol represents a promising physiological marker of perioperative stress, with growing evidence that links elevated cortisol to metabolic, cardiovascular, and neuropsychiatric risk profiles. However, there are substantial limitations regarding diurnal rhythm, anesthetic exposure, surgical magnitude, underlying illness, and testing methodology, which currently limit its reliability for routine perioperative risk assessment. To address these limitations, it is essential to develop standardized strategies for measuring cortisol and IL-6. Proposed steps include establishing consensus on the timing and conditions of cortisol sampling that account for diurnal variations and surgical contexts, standardizing assay techniques to reduce variability across laboratories, and defining how these biomarkers should be interpreted in relation to postoperative complications and recovery patterns. Prospective perioperative studies that incorporate standardized sampling protocols and clearly defined outcome measures are needed before cortisol-based strategies can be broadly adopted. 

Table 1 below summarizes stress-related biomarkers, outlining their clinical significance, associations with postoperative outcomes, and the proposed biological mechanisms supported by current evidence.

## 7. Psychological Stress During the Perioperative Period

Although surgical trauma represents a significant factor of physical stress, it is important to consider that the perioperative period involves considerable psychological stress. Preoperative anxiety is a frequent and clinically significant issue in surgical settings, with reported prevalence ranging widely from 11% to 80% [3]. The incidence varies according to gender, the criteria used, the surgical indication, and the patients’ level of education [64,65,66].

Patients may present with anxiety due to fear of death, anesthesia, uncertainty about recovery, or fear of the unknown [67]. For some patients, being in an unfamiliar hospital environment, feelings of loss of control, separation from family, and previous negative medical experiences can further amplify preoperative distress [68]. This psychological stressor triggers neuroendocrine changes, with activation of the HPA axis and characteristic increases in ACTH as well as cortisol during perioperative period [69,70].

Because of the higher risk of adverse events, the use of benzodiazepines for controlling pre-surgical anxiety has been decreasing [68] and other management strategies are emerging as effective and patient-centered alternatives [71]. Many strategies have been studied in an attempt to alleviate pre-surgical stress. The practice of offering information about surgical procedures, operating room processes, anatomy, and postoperative care protocols, among others, seems to reduce anxiety levels [72,73]. Music therapy, massages and virtual reality have proven to be effective, economically viable, minimally invasive and low-risk [74].

## 8. Depression, Anxiety and Neuropsychiatric Postoperative Outcomes

Depression, cognitive dysfunction and increased anxiety are very common in postoperative periods [75,76]. There are many proposed pathways that may be implicated in this relationship, but no direct mechanism has been identified. Disrupted circadian rhythm, inflammatory cytokines, and abnormal HPA axis signaling may all contribute to preoperative stress and are associated with postoperative psychologic symptoms [77,78,79,80].

IL-6 is a pro-inflammatory cytokine that increases during psychological stress and plays a role in regulating many immune and inflammatory processes [78,81]. It has a strong effect on the body’s stress response in the CNS and on the periphery [82]. It is released by neurons, astrocytes, and microglia in response to inflammation. It also has stimulatory effects that promote the release and activation of downstream inflammatory cytokines such as IL-1B and C-reactive protein (CRP) in the periphery. This widespread distribution of IL-6 has been demonstrated in rat models exposed to chronic stress, where IL-6 was found elevated in serum and striatum [83]. This elevation in serum IL-6 has been reproduced in human populations, and it has been proposed as a marker of chronic inflammation and depressed mood [81]. IL-6 levels have been reported to correlate with preoperative anxiety and postoperative depression and hostility in patients undergoing open heart surgical procedures [78]. Increased levels of IL-6 postoperatively were also significantly associated with postoperative delirium in a similar population [84]. These results have been reproduced in patients undergoing total knee replacement [85]. In mouse models, elevated IL-6 was associated with decreased social interaction and other depressive symptoms [86]. 

In addition to IL-6, IL-1b causes increased expression of inflammatory proteins in the brain and is commonly elevated in animal models of depression and chronic stress in rodents. Increased IL-1b has been associated with decreased social interaction, appetite and activity in animal models. It has been shown that IL-1b has increased expression during stress and is associated with increased reactive astrogliosis in mice brains associated with memory, suggesting a link between its expression and cognitive impairment. Additionally, depressive behavior in animal models has been shown to decrease when IL-1b receptor antagonists are administered [83].

C-reactive protein is another biomarker released primarily from the liver associated with inflammation that may be related to psychologic symptoms after surgery and trauma [82,87,88]. A correlation between depression and levels of CRP has also been found [87,88]. In patients undergoing knee surgery, postoperative levels of both CRP and IL-6 were positively correlated with depressive symptoms [83]. Additionally, higher levels of CRP and IL-6 on admission were both correlated with higher rates of delirium in an ICU population. Because IL-6 and CRP affect many different immune and inflammatory processes, it remains controversial in their direct involvement in the development of acute postoperative outcomes despite their correlation in some smaller observational studies [78].

Depression and pain have been linked and appear to be risk factors for each other [88,89]. Depression and anxiety have both been associated with increased pain ratings and a larger consumption of pain medications in the early postoperative period [90]. Additionally, depression and anxiety have been significantly correlated with an increased likelihood of various types of acute pain postoperatively such as headache, abdominal, chest and low back pain and other postoperative pain in a large database of patients undergoing spine surgery [91]. 

When both pain and depression are present together, their presentation tends to be more severe, suggesting their development may share a similar pathway [90]. One proposed mechanism is that patients suffering from anxiety and/or depression are at increased risk of interpreting novel sensations as pain [92]. Given that pain perception involves modulation from the central nervous system, it would be logical that conditions that affect the brain such as depression and anxiety would negatively affect processing of nociceptive stimuli as well [93]. Pain causes defective signaling in the prefrontal cortex, hippocampus, and ventral tegmental area, which are also implicated in the symptoms of depression [94]. Additionally, the serotonin signaling pathway may be implicated in depression and postoperative pain [95]. These acute changes may also affect the pain signaling pathway long-term. Depression, anxiety and the development of chronic pain has also been well established [90,93]. Chronic inflammation is strongly associated with the development of depression, indicating that there may be a peripheral component in the relationship between chronic pain and depression [94]. The interplay between inflammation, depression, and postoperative pain is complex, but their association may indicate that appropriate treatment in the acute setting may help prevent the development of chronic pain syndromes. 

Preoperative depression [96] contributes to the postoperative delirium development. Delirium during the postoperative period has been associated with increased medical costs [89]. Depression is commonly associated with cognitive impairment and the additional stress during the perioperative period may worsen cognition leading to postoperative delirium [90]. Conversely, patients who experience postoperative delirium are at increased risk of experiencing depression within 6 months of surgery [97]. This suggests that depression and delirium may affect similar signaling pathways in their development [98]. The body’s stress response during surgery, the activation of the HPA axis and inflammatory process may be implicated. The activation of both of these pathways can lead to altered cognition, mood, and motivation, leading to depressive symptoms and delirium [99]. This correlation may be a means to further stratify patients who are at risk of developing delirium and take precautions to prevent delirium in the postoperative setting.

Cortisol secretion is dependent on many factors, and can be influenced by type of anesthesia [84], type of surgery [79] and perioperative medications [100]. It has been shown that general anesthesia does not increase cortisol secretion as much as local anesthesia in sentinel lymph node excision [80]. Additionally, coronary artery bypass is commonly associated with postoperative delirium and depression [75]. Cardiac surgery has also been demonstrated to display a large and prolonged release of cortisol [79]. Commonly used drugs in anesthesia, propofol and opiates [30] are known to inhibit cortisol secretion. Depending on the interplay of these factors, the pulsatile release of cortisol can be altered, leading to changes in mood, attention and circadian rhythm [79].

In patients with depression, disruption of cortisol secretion caused reduced scoring in tests involved in short-term memory, executive function and visual perception [77,79]. This suggests that patients with anxiety or depression may have a dysregulated HPA axis leading to abnormal levels of cortisol, and may be more sensitive to the homeostatic disturbances associated with the perioperative state [101,102]. This may lead to further downstream signaling that leads to continued inflammation in the CNS and atrophy of the hippocampus, increasing risk for memory dysfunction [103,104]. Further research in determining the effectiveness of interventions to reduce disturbances in HPA axis signaling in patients with depression would be useful to prevent perioperative cognitive dysfunction.

In summary, how stress and inflammation in the perioperative period affects psychologic conditions such as pain, delirium, and depression is complex and multifactorial. Inflammatory proteins that are easily measured in the serum, such as IL-6, IL-1b, CRP, and cortisol, show promise as risk factors for developing perioperative psychological conditions as they are correlated with increased stress, delirium, pain, and depression. However, their direct role in the development of these conditions in the perioperative period is controversial and limits their application to clinical practice due to the many factors that can affect their release. Further research is needed to demonstrate the strength of their relationship with psychological conditions in the perioperative period, as well as the effectiveness of interventions aimed at targeting these inflammatory signals.

## 9. Biological Links Between Stress and Postoperative Infection

Surgical stress increases the risk of postoperative infections (POIs). A key mediator in this process is hyperglycemia, which is a common metabolic response to perioperative stress that impairs the host defenses [105]. The immune response to stress is characterized by upregulation of pro-inflammatory and anti-inflammatory cytokines, including IL-6, TNF-α and IL-1β, as well as anti-inflammatory cytokines like IL-10 and TGF-β [106]. These molecules are regulated through signaling pathways like NF-κB, MAPK, and JAK/STAT which modulate tissue repair, leukocyte recruitment and systemic inflammatory response perioperatively.

However, in a state of acute stress and elevated glucose, namely perioperatively, IL-6 levels have been amplified [107]. Elevated IL-6 levels may serve as a predictive biomarker, as it has been proposed as a useful marker in the prediction of postoperative complications, including infections [108].

Hyperglycemia activates multiple metabolic pathways, including polyol and hexosamine pathways, which increase levels of ROS and depletes NAD+/NADPH stores, leading to oxidative stress and subsequent NF-κB activation [109]. Additionally, hyperglycemia increases DAG levels, activating protein kinase C (PKC), an upstream modulator of NF-κB signaling [110]. Both NF-κB and AP-1 bind to IL-6 promoter sites in endothelial cells, monocytes, adipocytes, and other immune cells, upregulating IL-6 transcription. Once IL-6 binds to its membrane-bound IL-6 receptor alpha chain (mIL-6R), it induces homodimerization of the gp130 which initiates the JAK1/2-STAT3 pathway [111]. This phosphorylated STAT3 translocates to the nucleus to promote transcription of CRP and CXCL8 [112,113,114]. Longer duration of stress promotes leukocyte recruitment via increased endothelial adhesion molecule expression, such as VCAM-1 and ICAM-1, ultimately playing a role in immune response and infection risk [113]. 

For both diabetic and non-diabetic patients undergoing surgical procedures, it was found there was an increase in blood glucose levels in response to stress. Postoperative hyperglycemia (postoperative glucose from 110 to 200) increased the risk of 30-day POI rate by 30% and a longer hospital stay [115]. Hyperglycemia impairs key immune responses like neutrophil chemotaxis, production of ROS, and blunt vasodilation, all critical responses which increase the risk of infection due to defective immune response. 

Acute hyperglycemia has also been found to cause diminished vasodilation. Although the precise mechanism is complex and not fully understood, current theory suggests that hyperglycemia increases oxidative stress in the vascular endothelium leading to excess ROS production. This excess ROS is theorized to scavenge NO into peroxynitrite, which directly reduces NO bioavailability but also potential oxidation of BH4, which can cause endothelial NO synthase (eNOS) uncoupling and further ROS production, all while reducing NO levels [116]. 

Although the direct link between impaired vasodilation and risk of infection remains underexplored, vascular endothelium plays a central role in immune cell trafficking, suggesting a potential link to infection risk. A functional vascular epithelium is needed for proper defenses against infections, an example includes ICAM-1 and VCAM which are normally important players in diapedesis, a mechanism by which immune cells enter the tissue. However, in the setting of inflammation, these factors can be impaired leading to decreased immune defenses at the tissue site increasing susceptibility to postoperative infections [117].

In the setting of hyperglycemia, a study found that physiologic levels of insulin do not have a profound effect on blood flow. However, it was found that prolonged insulin transfusion can cause vasodilation via two interconnected mechanisms: PI3-kinase cascade and stimulation of Na^+^/K^+^ ATPase [118].

One mechanism of insulin-mediated vasodilation includes PI3-kinase which is an intracellular enzyme that is activated due to upstream insulin binding to an endothelial receptor. After insulin binds to its receptor in the endothelium, the downstream PI3-kinase generates phosphatidyl-inositol-(3,4,5)-trisphosphate (PIP3) which then recruits Atk and subsequently phosphorylates eNOS, which increases nitric oxide production, resulting in vasodilation. This mechanism is further supported by the addition of Wortmannin which inhibits PI3-kinase, which causes about a 50% decrease in the insulin effect and downstream vasodilation [119,120].

There are two distinct possible sites of action of insulin and Na^+^/K^+^ ATPase involving the smooth muscle cells and endothelial cells. Regarding the smooth muscle cells, insulin stimulates and activates the Na^+^/K^+^ ATPase, hyperpolarizing the cell. This hyperpolarization closes voltage-dependent calcium channels, thus decreasing the intracellular Ca2^+^ concentration, resulting in relaxation and vasodilation. Another proposed mechanism involves action at the endothelial cells where the insulin stimulating the Na^+^/K^+^ ATPase does not close the Ca2^+^ channels because they are voltage-independent; instead, it increases the electrochemical gradient to allow more Ca2^+^ to enter the cell. Increased intracellular Ca2^+^ activates eNOS, increasing NO release; the NO diffuses to smooth muscle and causes vasodilation. The idea that insulin directly affects Na^+^/K^+^ ATPase and causes vasodilation is further supported by the addition of Ouabain (an inhibitor of Na^+^/K^+^ ATPase) [120]. 

Taken together, these findings highlight that when insulin is administered for 4–6 h, it can cause vasodilation via the PI3-kinase cascade and stimulation of Na^+^/K^+^ ATPase, which converges on NO production, resulting in vasodilation. Given that diminished vasodilation in the setting of acute hyperglycemia can impair cellular function and the systemic immune response which can increase infection risk, these findings offer clinically relevant implications. Specifically, tight glucose control and insulin therapy preoperatively and postoperatively may help restore vascular function and microcirculatory perfusion and could plausibly reduce susceptibility to infection, although direct evidence in stress-stratified surgical cohorts remains limited. These insulin-mediated vascular effects warrant further prospective study before being considered established strategies for preventing postoperative infection. 

Current evidence supports a biologically plausible link between perioperative stress, hyperglycemia, endothelial dysfunction, and impaired immune response. Clinical cohort studies associate hyperglycemia with an increased risk of infection, but the extent to which stress-targeted or insulin-based interventions specifically reduce infections remains underexplored [115]. Optimizing glucose control and vascular function may therefore represent important strategies to mitigate postoperative infection risk. However, this hypothesis has not yet been tested in trials that consider baseline psychological stress. Prospective studies integrating standardized stress assessments, perioperative glucose patterns, and infection outcomes are needed to determine whether metabolic interventions based on preoperative stress levels reduce postoperative infection risk.

## 10. Stress, Cortisol and Postoperative Pain

Effective postoperative pain management is very important for reducing the consequences of acute postoperative pain and facilitating smooth recovery [121]. Inadequately controlled pain is associated with anemia, hypoxia, and prolonged sympathetic activation, which together increase cardiovascular risk in the postoperative period [122]. Consequently, identifying patients at higher risk for severe postoperative pain represents a critical step in perioperative optimization. Multiple psychological and biological factors have been implicated in this process, including preoperative anxiety and depression, pain catastrophizing, individual pain sensitivity, as well as baseline opioid consumption [123,124].

Stress-related hormonal alterations may have an important role in shaping postoperative pain perception. Acute psychological stress is consistently associated with elevations in cortisol and concomitant suppression of testosterone, a hormonal profile that has been linked to increased pain sensitivity and reduced pain thresholds [125]. These findings support the concept that stress enhances nociceptive processing through endocrine mechanisms, rather than through subjective distress alone.

Beyond experimental settings, cortisol has also emerged as a potential objective marker of pain intensity in real-world clinical contexts. Observational data from large prehospital cohorts demonstrate a strong association between circulating cortisol levels and NRS scores, particularly in patients experiencing moderate to severe pain [126]. This relationship appears less pronounced in cases of mild pain, suggesting that cortisol may reflect the biological intensity of the stress–pain response rather than pain perception per se. The direction of stress-induced pain modulation is not uniform across individuals. While stress is often associated with hyperalgesia, controlled experimental stress paradigms have shown that stress-induced hypoalgesia may occur in individuals who exhibit a robust cortisol response [127]. In such cases, activation of the HPA axis, rather than sympathetic arousal alone, appears to mediate transient increases in pain thresholds, highlighting the complexity and context-dependence of stress–pain interactions.

Clinical perioperative studies further underscore the bidirectional relationship between analgesia, stress responses, and recovery. Enhanced analgesic strategies, particularly those incorporating regional techniques, reduce postoperative pain scores while simultaneously attenuating cortisol release and shortening intensive care unit stays [128]. All of this suggests that effective pain control may not only improve subjective comfort but also blunt the neuroendocrine stress response, thereby contributing to improved postoperative outcomes.

Despite these mechanistic and observational links, the relationship between preoperative anxiety and postoperative pain remains heterogeneous at the population level. Large-scale meta-analytic data have failed to demonstrate a consistent association between anxiety and pain outcomes across surgical procedures, likely reflecting substantial variability in surgical type, analgesic protocols, and study methodology [5]. This heterogeneity shows the need for individualized risk stratification rather than reliance on single psychological predictors.

## 11. Stress-Related Pain Outcomes and Modulation

Preoperative psychological stress, particularly anxiety, is a highly prevalent and clinically significant factor in pain outcomes. Epidemiological evidence indicates that preoperative anxiety affects approximately 60–80% of surgical patients, reflecting the substantial psychological burden preceding surgery [5]. This high prevalence of anxiety is epidemiologically relevant, as psychological distress has been repeatedly linked to adverse pain outcomes. For chronic postsurgical pain (CPSP), about 55% of studies report a significant correlation in elevated preoperative anxiety or pain catastrophizing and the subsequent development of CPSP [129]. Preoperative anxiety is also associated with an overall increase in chronic pain intensity, reflected as an approximate 0.34 point rise on the NRS for patients with higher anxiety levels [130]. In specific surgical populations, such as older patients undergoing off-pump coronary artery bypass grafting, 37.8% developed CPSP at 3 months, with preoperative anxiety present in 46.2% of these patients, and anxiety independently predicted CPSP [131]. Preoperative anxiety also influences acute postoperative pain responses: prospective studies demonstrate that patients with heightened preoperative anxiety have significantly higher postoperative pain scores and greater analgesic consumption in the immediate postoperative period than low-anxiety counterparts, underlining how stress impacts both the severity and duration of pain after surgery [6].

Stress-related modulation of pain manifests not only through neurobiological mechanisms but also via measurable clinical effects, with a high burden of anxiety, preoperatively increasing the risk for both acute and chronic pain sequelae.

## 12. Conclusions

Preoperative stress represents a highly prevalent yet underrecognized risk factor that influences surgical outcomes. Across various biological pathways, this review emphasizes a unified theme: stress is not a benign emotional state but rather a biologically potent modifier of perioperative physiology. As highlighted, stress-driven activation of the HPA axis, elevations in cortisol, and increased pro-inflammatory cytokine release (particularly IL-6) creates a cascade that impairs immune function, pain processing, and metabolic homeostasis. These physiological changes are associated with outcomes commonly encountered in perioperative practice, including higher analgesic and anesthetic requirements, increased rates of postoperative delirium, elevated infection risk, and longer hospital stays. 

Despite the clear associations, the mechanistic links connecting preoperative stress and postoperative outcomes remain insufficiently explored. Current evidence is fragmented; biomarkers like cortisol and IL-6 are promising, yet they lack standardized perioperative interpretation.

Importantly, preoperative stress is modifiable. Emphasis on psychological interventions, structured patient education, optimized analgesic and glucose management can reduce the stress response and improve postoperative outcomes. Identifying high-risk patients through stress screening or biomarkers, such as increased cortisol or IL-6, may allow for earlier intervention and customized perioperative planning. 

This review highlights the potential value of incorporating preoperative stress assessment as a component of preoperative evaluation and using this information to tailor interventions to each patient. By recognizing preoperative stress as a potentially important modifier of perioperative risk, anesthesia and surgical teams may ultimately be able to reduce complications, enhance recovery, and deliver more patient-centered care, pending validation in prospective trials.

## Figures and Tables

**Table 1:** Stress-related biomarkers and their mechanistic links to postoperative outcomes.

Biomarker	Clinical Significance	Postoperative Implications	Mechanism
Cortisol	HPA axis activation, neuroendocrine stress response	↑ Pain sensitivity, ↑ delirium risk, cognitive dysfunction, ↑ metabolic complications	Glucocorticoid receptor signaling modulates inflammation (NF-κB), disrupts circadian rhythm, alters CNS excitability and energy regulation
IL-6	Pro-inflammatory cytokine, acute phase response	↑ Risk of delirium, ↑ depressive symptoms, ↑ infections, ↑ pain	Induces CRP, activates JAK/STAT3 signaling, crosses BBB, modulates hippocampal and limbic function
CRP	Acute inflammatory marker, systemic stress responder	↑ Depression scores, ↑ infection risk	Hepatic release via IL-6, downstream of NF-κB activation; linked to neuroinflammation and endothelial dysfunction
IL-1β	Neuroimmune modulator, stress-induced neuroinflammation	↓ Social interaction, ↑ risk of postoperative depression/delirium	Activates microglia, promotes astrogliosis and hippocampal inflammation; reversible via IL-1β antagonists in animal models
Glucose	Stress-induced hyperglycemia, metabolic stress marker	↑ Infection risk, ↑ endothelial dysfunction, ↑ hospital stay	Activates ROS → NF-κB → ↑ IL-6; reduces nitric oxide bioavailability via eNOS uncoupling, promotes leukocyte adhesion via ICAM-1/VCAM-1
Salivary cortisol	Noninvasive stress biomarker, correlates with plasma cortisol	Potential perioperative monitoring tool; predictive of acute stress and pain	HPA axis marker; pulsatile secretion altered by anesthesia, circadian rhythm, psychological state

Abbreviations: HPA, hypothalamic–pituitary–adrenal axis; CNS, central nervous system; NF-κB, nuclear factor kappa B; IL-6, interleukin-6; CRP, C-reactive protein; JAK/STAT3, Janus kinase/signal transducer and activator of transcription 3; BBB, blood–brain barrier; IL-1β, interleukin-1 beta; ROS, reactive oxygen species; eNOS, endothelial nitric oxide synthase; ICAM-1, intercellular adhesion molecule-1; VCAM-1, vascular cell adhesion molecule-1.

## Data Availability

No new data were created or analyzed in this study.
